# Genetics of evolved load resistance in the skeletons of unusually large mice from Gough Island

**DOI:** 10.1093/genetics/iyad137

**Published:** 2023-07-21

**Authors:** Bret A Payseur, Sara Anderson, Roy T James, Michelle D Parmenter, Melissa M Gray, Christopher J Vinyard

**Affiliations:** Laboratory of Genetics, University of Wisconsin-Madison, Madison, WI 53706, USA; Department of Anatomy and Neurobiology, Northeast Ohio Medical University, Rootstown, OH 44272, USA; Department of Anatomy and Neurobiology, Northeast Ohio Medical University, Rootstown, OH 44272, USA; Laboratory of Genetics, University of Wisconsin-Madison, Madison, WI 53706, USA; Laboratory of Genetics, University of Wisconsin-Madison, Madison, WI 53706, USA; Department of Biomedical Sciences, Ohio University - Heritage College of Osteopathic Medicine, Athens, OH 45701, USA

**Keywords:** skeleton, load resistance, bone geometry, island rule

## Abstract

A primary function of the skeleton is to resist the loads imparted by body weight. Genetic analyses have identified genomic regions that contribute to differences in skeletal load resistance between laboratory strains of mice, but these studies are usually restricted to 1 or 2 bones and leave open the question of how load resistance evolves in natural populations. To address these challenges, we examined the genetics of bone structure using the largest wild house mice on record, which live on Gough Island (GI). We measured structural traits connected to load resistance in the femur, tibia, scapula, humerus, radius, ulna, and mandible of GI mice, a smaller-bodied reference strain from the mainland, and 760 of their F2s. GI mice have bone geometries indicative of greater load resistance abilities but show no increase in bone mineral density compared to the mainland strain. Across traits and bones, we identified a total of 153 quantitative trait loci (QTL) that span all but one of the autosomes. The breadth of QTL detection ranges from a single bone to all 7 bones. Additive effects of QTL are modest. QTL for bone structure show limited overlap with QTL for bone length and width and QTL for body weight mapped in the same cross, suggesting a distinct genetic architecture for load resistance. Our findings provide a rare genetic portrait of the evolution of load resistance in a natural population with extreme body size.

## Teaser

Payseur *et al*. characterize the genetics of evolved differences in bone structure between mice from Gough Island (the largest wild house mice on record) and mice from the mainland. They identify many genomic regions that contribute to these differences, ranging in their potential effects from one bone to several bones. Their findings suggest that Gough Island mice evolved increased load resistance abilities through a series of modest genetic steps.

## Introduction

The skeleton supports the body, protects vital organs, and facilitates locomotor movement. Bone stiffness and strength are crucial to these functions. Bones must resist deformation (stiffness) and failure (strength) to handle the loads caused by bearing weight and performing movement (Jepsen 2009).

The ability of bone to withstand mechanical loads is determined by its material and structural properties (Hart *et al*. 2017). On the material side, the quality of bone is primarily influenced by its mineral density, which is the product of mineralization and porosity (Hart *et al*. 2017). From the structural perspective, cross-sectional geometry is a key determinant of a bone's ability to resist loads (Adams and Ackert-Bicknell 2015). The most relevant morphological parameter for loading in compression (pushing) or tension (pulling) is a cross-sectional cortical bone area, whereas the cross-sectional moment of inertia describes the potential for resistance to bending and torsion (Jepsen *et al*. 2015).

Inbred strains of mice raised in the same laboratory environment differ in structural and material properties of long bones (Beamer *et al*. 1996; Jepsen *et al*. 2003; Wergedal *et al*. 2005; Reich *et al*. 2008), demonstrating the contribution of genetic factors and positioning mice as a powerful experimental system for characterizing them. Suites of quantitative trait loci (QTL) shape variation among mouse strains in bone strength, cross-sectional geometry, and mineral density (Beamer *et al*. 2001, 2012; Klein *et al*. 2002; Adams and Ackert-Bicknell 2015; Lu *et al*. 2019). QTL for bone geometry and material composition sometimes colocalize with QTL for whole-bone mechanical strength evaluated in functional tests (Jepsen 2009; Adams and Ackert-Bicknell 2015) and with QTL for obesity (Reich *et al*. 2008), providing genetic confirmation of the relevance of bone structure for load resistance. Fine-mapping and systems genetic analyses have identified candidate genes for traits involved in load resistance, including *Qsox1*, which controls cortical bone mass and strength in diversity-outbred mice (Al-Barghouthi *et al*. 2021).

Despite this progress, 2 important questions remain largely uninvestigated in the genetic characterization of load resistance abilities. First, are the structures that determine load resistance abilities in different bones generated by the same set of loci? Although mechanical and developmental considerations suggest potential for distinct genetic architectures across the skeleton (Hall 2005), most genetic mapping studies of bone strength focus on the femur (or the femur and the tibia), leaving this question unaddressed. Second, what is the genetic basis of evolved differences between natural populations in bone structures that resist load? Genetic mapping studies to date are restricted to classical inbred mouse strains and their derivatives, leaving open the question of how bone structure adapts to increases in load in response to novel environments in the wild.

Natural populations that have recently evolved substantial increases in body size are promising targets for the genetic investigation of skeletal load resistance abilities. Mice on Gough Island (hereafter “GI”), a remote island located near the middle of the South Atlantic Ocean, are the largest wild house mice on record (Rowe-Rowe and Crafford 1992; Gray *et al*. 2015), weighing close to twice as much as their mainland relatives (Jones *et al*. 2003; Gray *et al*. 2015). This remarkable expansion in size likely happened during the few hundred generations following the colonization of the island (Rowe-Rowe and Crafford 1992). GI mice stand as a prominent example of the island rule, a taxonomically broad pattern among vertebrates in which island populations evolve extreme body sizes compared to their mainland counterparts (Foster 1964; Van 1973; Lomolino 1985; Benítez-López *et al*. 2021). The bones of GI mice must resist the additional load imposed by their extra body weight, which could have led to the evolution of bone structural and/or material properties in these mice. GI mice belong to the same subspecies as classical inbred strains (the Western European house mouse, *Mus musculus domesticus*) (Gray *et al*. 2014), facilitating the genetic examination of skeletal traits. In previous work, we uncovered the genetic basis of evolution in body size and skeletal shape in GI mice (Gray *et al*. 2015; Parmenter *et al*. 2016, 2022).

In this study, we use GI mice as a model system to understand how morphological determinants of skeletal load resistance abilities evolve in nature. We identify QTL that contribute to differences between GI mice and a mainland reference strain in the structures of 7 bones. Our findings extend genetic understanding of load resistance across the skeleton and provide clues into the changes that accompany the evolution of extreme body size.

## Materials and methods

### Mice

GI is part of the United Kingdom Overseas Territory of Tristan da Cunha located in the South Atlantic Ocean, approximately halfway between South America and South Africa (40° 19′S and 9° 55′W). Fifty mice live-trapped on GI in September 2009 were transferred to the Charmany Instructional Facility in the School of Veterinary Medicine at the University of Wisconsin-Madison. Upon their arrival, 46 mice (25 female and 21 male) were used to establish a breeding colony.

All mice in this study were housed at the University of Wisconsin-Madison Charmany Instructional Facility (Madison, WI). Female mice and male mice were housed separately in micro-isolator cages with a maximum of 4 mice per cage. Ground corn cobs (1/8th inch; Waldschmidt and Sons, Madison, WI) were used as bedding; nesting material and irradiated sunflower seeds (Harlan Laboratories, Madison, WI) were provided for enrichment. The room was temperature controlled (68–72°F) and set on a 12-hour light/dark cycle. Water and rodent chow (Teklad Global 6% fat mouse/rat diet; Harlan Laboratories, Madison, WI) were provided ad libitum. Breeding individuals were fed breeder chow (Teklad Global 19% protein/9% fat; Harlan Laboratories, Madison, WI) ad libitum. All mice were weaned between 3 and 4 weeks of age.

Two partially inbred lines of GI mice were generated through full-sib mating for 4 filial generations (Gray *et al*. 2015; Parmenter *et al*. 2016). Mice from the fully inbred, wild-derived strain WSB/EiJ (subsequently “WSB”) were ordered from Jackson Laboratories (Bar Harbor, ME), mated to generate mice born in our facility, and treated as a mainland reference for comparisons with GI mice.

To characterize phenotypic differences between GI mice and WSB mice, we separately constructed a linear model for each trait, with strain and sex treated as fixed effects. Sample sizes for these comparisons were 30 GI mice (17 females; 13 males) and 24 WSB mice (13 females; 11 males).

One pair of female and male siblings from each of 2 partially inbred lines of GI mice was crossed with WSB. F1s were intercrossed to generate F2s. Altogether, 4 independent F2 intercrosses were performed (Gray *et al*. 2015; Parmenter *et al*. 2016). Genetic analyses of bone traits focused on 760 F2 mice: 405 from Cross A (WSB × GI = 243; GI × WSB = 162) and 355 from Cross B (WSB × GI = 203 and GI × WSB = 152). All F2 mice were weighed at 16 weeks of age and euthanized by CO_2_ asphyxiation or by decapitation. This study was approved by the Institutional Animal Care and Use Committee at the University of Wisconsin-Madison.

### Phenotyping

Our study focused on limb bones including the femur, tibia, scapula, humerus, radius, and ulna, as well as the mandible. We did not survey the pelvis because identifying a cross-sectional middle is more challenging. After dissecting away soft tissues, skeletal elements were scanned by micro-computed tomography (micro-CT) at a 20.5 μm voxel resolution in a vivaCT 70 scanner (Scanco) at 70 kVp/114 mA. We output scans as stacked 2,048 × 2,048 tiff images. These images were cropped for each skeletal element in Adobe Photoshop. We imported stacked tiff images into Avizo where digital reconstructions of skeletal elements were resliced at the diaphyseal midpoint for long bones, the scapular neck, and the mandibular M1 for cross-sectional analysis. Resliced tiff images were imported into ImageJ2 (Fiji) (Rueden *et al*. 2017) for cross-sectional analysis using BoneJ (Doube *et al*. 2010).

**Fig. 1.**
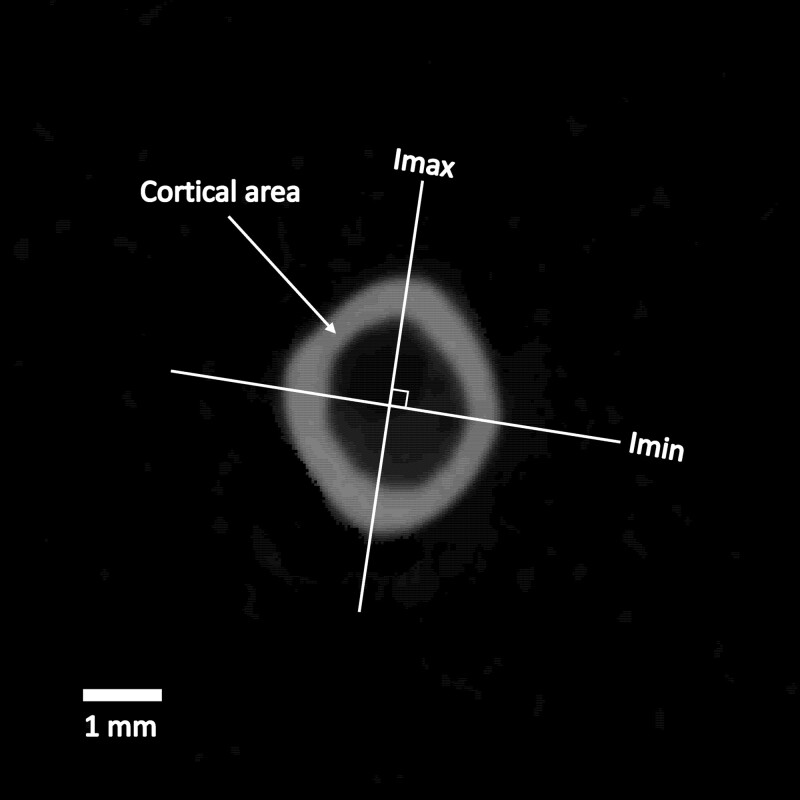
Structural measurements of bone. A micro-CT image from a cross-section of the femur of a GI mouse is shown. Cortical area is estimated as the summed areas of bone in cross-section (lighter shade). Lines indicate orientations for calculating maximum area moment of inertia (Imax) and minimum area moment of inertia (Imin). Polar moment (J) is computed as Imax + Imin.

We characterized cross-sectional properties of bones related to load resistance abilities using 4 standard measurements (Fig. 1). Cortical area (CA), an indicator of the ability to resist shear, was measured as the total area of bone in a cross-section. Imax, the maximum area moment of inertia and an indicator of maximum resistance to bending, was measured as the sum of bone area times its squared distance from the bone centroid. Imin, the minimum area moment of inertia and an indicator of minimum resistance to bending, was measured similarly and is perpendicular to Imax. J, the polar moment of inertia and an indicator of resistance to torsion, was measured as the sum of Imax and Imin. Our genetic analyses considered CA, Imax, Imin, and J collected from each of the 7 bones analyzed.

Since differences in bone quality between GI mice and WSB mice would impact the interpretation of bone cross-sectional data, we compared bone density in these 2 groups. Bone density was calculated by converting grayscale values from bone cross-sections to mg/HA based on a linear regression equation created from known calibrations between grayscale values and mg/HA using manufacturer-supplied phantoms (R = 0.999): mgHA/cm^3^= −222.813 + 6.927 (grayscale value).

### Genotyping

Genotypes for QTL mapping were taken from Gray *et al*. (2015). Briefly, livers from F2s, GI parents of the cross, and WSB parents of the cross were sent to Geneseek (NeoGene Corporation) for DNA extraction and genotyping. Mice were genotyped using the Mega Mouse Universal Genotyping Array (MegaMUGA; Geneseek, Lincoln, NE), an Illumina array platform containing 77,800 single nucleotide polymorphisms (SNPs), evenly spaced throughout the genome (Threadgill and Churchill 2012). After data cleaning, analyses focused on 11,833 fully informative SNPs that were fixed in the 4 GI mouse parents and therefore segregated as in a standard F2 intercross between inbred lines. We estimated genetic distances between SNPs assuming a genotyping error rate of 0.2% and the Carter–Falconer mapping function (Carter and Falconer 1951). The genetic map was reconstructed using 1,212 F2s (Gray *et al*. 2015).

### QTL mapping and characterization

To identify QTL for each trait separately, we first conducted a single-QTL scan, which could detect up to one QTL per chromosome, using Haley–Knott regression in the *scanone* function from the R/qtl package (Broman *et al*. 2003). A QTL was deemed significant if its logarithm of odds (LOD) score exceeded the genome-wide 5% threshold set by 1,000 permutations (Churchill and Doerge 1994). Next, we performed a multiple-QTL scan with a stepwise search using a penalized LOD score as the criterion (Broman and Speed 2002), implemented by *stepwise* in R/qtl. We initialized the search with QTL detected by single-QTL mapping, used the significance threshold from single-QTL mapping as the penalty, and assumed QTL effects combine additively across loci. Based on results from single-QTL scans, we set the maximum number of possible QTL at 10 for all traits except for the humerus, for which we set the maximum number of QTL at 15. Additive effects of QTL (*a*) were calculated as half the difference between genotypic means of GI homozygotes and WSB homozygotes and scaled by dividing by the F2 phenotypic standard deviation for the trait of interest. Dominance effects (*d*) were calculated as the difference between the genotypic mean of GI/WSB heterozygotes and the midpoint of the genotypic means of the 2 homozygotes and scaled by dividing by the additive effect (*d*/*a*). Standard errors for *d*/*a* were computed using the delta method (Lynch and Walsh 1998). The total percent of F2 variance explained by QTL was calculated from the full model. The percent of F2 variance explained by individual QTL was computed by comparing the full model to a sub-model without that QTL. QTL effects were estimated using *fitqtl* in R/qtl.

To map QTL while accounting for correlations among traits, we conducted principal component analyses (PCAs) of phenotypes across F2s. For these analyses, we excluded all measurements from the mandible and Imin measurements from all bones to reduce redundancy and focus on the postcranial skeleton, leaving 18 measurements from across 6 bones. We computed principal components from the pairwise correlation matrix of these 18 traits using *princomp* in R. We separately used scores for principal components 1 and 2 as traits in single-QTL and multiple-QTL scans.

All QTL analyses included sex and mother as additive covariates. After preliminary single-QTL scans revealed limited evidence for the effects of the X chromosome, QTL analyses were restricted to the autosomes. We estimated 1.5 LOD drop intervals for all QTL using *lod.int* in R/qtl. To estimate genomic positions of QTL, we found the locations of the closest genotyped SNPs in the grcm39 version of the mouse reference genome (using updates kindly provided by Karl Broman: https://raw.githubusercontent.com/kbroman/MUGAarrays/master/UWisc/mm_uwisc_v2.csv).

For a subset of QTL with overlapping confidence intervals across many traits, we compared the fit of a null model assuming a single QTL affects the set of traits to the fit of models assuming 2 linked QTL affect the set of traits. This analysis was implemented in the *qtlpvl* package in R/qtl using the “testpleio.1vs2” command with the “complete” search method and 100 parametric bootstrap simulations to estimate *P*-values under the null model of a single QTL.

We searched for candidate genes for QTL that affect many load resistance traits. First, we re-estimated the QTL position by examining all traits associated with the QTL using the “scanone.mvn” command in the *qtlpvl* package. In Mouse Genome Informatics (Ringwald *et al*. 2022), we searched the 1.5-LOD interval for genes associated with “abnormal limb long bone morphology” (MP: 0011504) and examined the resulting references for reports of bone phenotypes. We also searched the interval for genes with nonsynonymous differences between GI mice and WSB mice used in the cross. For this purpose, we applied *Liftover* in the UCSC Genome Browser (http://genome.ucsc.edu) to locate the 1.5-LOD interval in the grcm38 assembly. We used custom tracks in the University of California Santa Cruz (UCSC) Genome Browser we created from genome sequences of the partially inbred GI mice and WSB mice (Nolte *et al*. 2020) used as parents of the cross and the Variant Annotation Integrator (UCSC) to identify all nonsynonymous differences in the QTL interval that are fixed between these GI mice and WSB mice. We used the Variant Table in Ensembl to predict the deleteriousness of these nonsynonymous changes with the appproach called Sorting Intolerant from Tolerant (SIFT; https://ensembl.org/info/genome/variation/prediction/protein_function.html).

## Results

### Higher skeletal load resistance in GI mice compared to mainland reference strain

Analysis of variance (ANOVA) treating strain and sex as fixed effects factors indicates that most structural traits have significantly higher values in GI mice vs WSB mice (Table 1). Relative differences are generally higher for Imax, Imin, and J than for CA. Differences vary among bones, with traits from the scapula, humerus, and mandible tending to show higher relative values. ANOVA indicates that the sexes do not differ significantly for most structural traits in GI mice and WSB mice (Table 1). One exception is the mandible, where measurements tend to show higher values in females (Table 1).

**Table 1.** Effects of strain and sex on bone structure.

Bone	Trait	GI mean	WSB mean	Difference	Difference (%)	*F* strain (df = 1.49)	*P* strain	*F* sex (df = 1.49)	*P* sex
Femur	CA	0.875	0.788	0.088	11.13	3.94	0.052	2.25	0.14
Femur	Imax	0.222	0.143	0.079	55.13	19.68	5.18E−05	0.025	0.88
Femur	Imin	0.157	0.101	0.056	55.24	22.41	1.91E−05	3.14	0.08
Femur	J	0.379	0.244	0.135	55.18	22.08	2.15E−05	0.68	0.41
Tibia	CA	0.652	0.599	0.053	8.83	2.16	0.15	1.38	0.25
Tibia	Imax	0.084	0.055	0.029	53.36	17.26	1.30E−04	0.34	0.56
Tibia	Imin	0.059	0.045	0.014	31.86	10.03	0.003	0.62	0.44
Tibia	J	0.143	0.099	0.044	44.38	14.86	3.30E−04	0.004	0.95
Scapula	CA	0.686	0.448	0.238	53.15	78.72	9.13E−12	5.34	0.03
Scapula	Imax	0.135	0.067	0.068	100.70	87.5	1.76E−12	0.34	0.56
Scapula	Imin	0.037	0.021	0.016	76.68	44.17	2.34E−08	0	0.99
Scapula	J	0.172	0.088	0.084	94.95	77.95	1.06E−11	0.2	0.65
Humerus	CA	0.655	0.530	0.125	23.62	23.32	1.37E−05	1.7	0.2
Humerus	Imax	0.093	0.048	0.045	94.19	73.76	2.44E−11	0.63	0.43
Humerus	Imin	0.062	0.033	0.029	89.73	85.19	2.68E−12	3.05	0.09
Humerus	J	0.156	0.081	0.075	92.52	82.36	4.55E-−12	1.43	0.24
Radius	CA	0.309	0.269	0.040	14.88	8.88	0.005	3.72	0.06
Radius	Imax	0.016	0.008	0.008	94.30	36.57	1.98E−07	2.58	0.11
Radius	Imin	0.008	0.006	0.002	27.32	7.3	0.009	0.31	0.58
Radius	J	0.024	0.014	0.009	65.31	28.57	2.35E−06	1.86	0.18
Ulna	CA	0.316	0.276	0.040	14.39	10.78	0.002	1.29	0.26
Ulna	Imax	0.021	0.017	0.003	17.93	3.16	0.08	0.1	0.75
Ulna	Imin	0.006	0.003	0.003	89.85	38.6	1.10E−07	2.98	0.09
Ulna	J	0.026	0.021	0.006	28.86	8.15	0.006	0.01	0.92
Mandible	CA	2.027	1.494	0.534	35.72	74.87	1.95E−11	13.1	6.99E-04
Mandible	Imax	1.519	0.825	0.695	84.24	72.56	3.11E−11	6.32	0.02
Mandible	Imin	0.358	0.301	0.058	19.22	0.8	0.37	3.78	0.06
Mandible	J	1.878	1.040	0.838	80.51	89.66	1.19E−12	8.44	0.005

*F*-statistics and *P*-values from ANOVA.

ANOVA also demonstrates that strains do not differ significantly in the density of any of the 7 measured bones (*P* > 0.05 for all bones). Females and males differ significantly in the density of the mandible (*P* = 0.007), scapula (*P* = 0.004), radius (*P* = 0.048), and humerus (*P* = 0.035), with females having higher mean densities for each bone.

### Phenotypic correlations among F2s

**Fig. 2.**
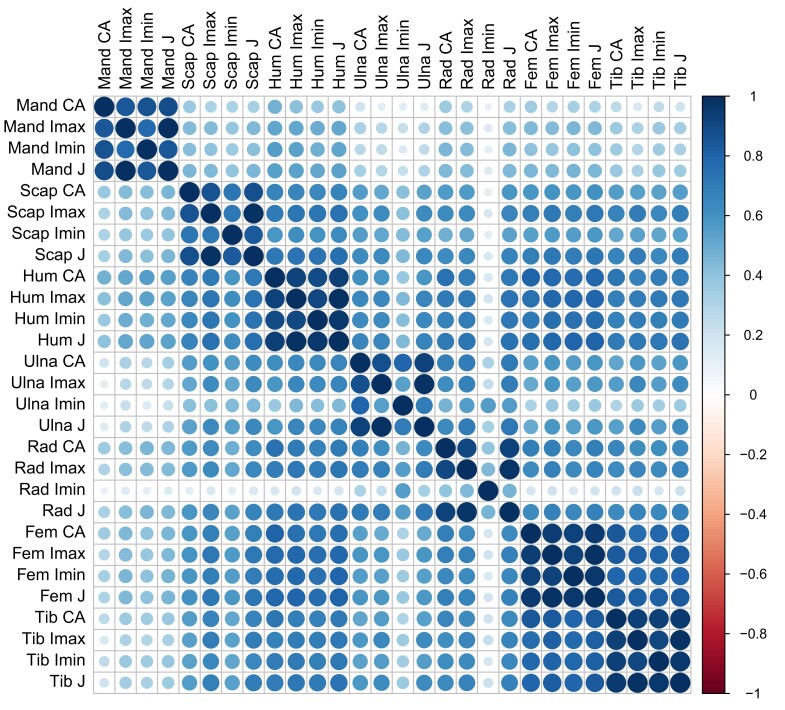
Pairwise correlations between bone structural traits across F2s. Magnitudes of Pearson's correlations are indicated by color and size of circles. *n* = 760 F2s.

Pairwise trait correlations across F2s show several patterns (Fig. 2). Pearson's correlations are universally positive with low *P*-values (all *P* ≤ 0.004). Different traits within bones are usually more highly correlated than the same measurements across bones. Femur with tibia and femur with humerus show the strongest correlations among bones. Correlations between the scapula (Imax and J) and femur, tibia, and humerus are also high. Overall, J traits have the highest correlations, followed by Imax, CA, and Imin. The weakest correlations involve the mandible or radius Imin.

**Fig. 3.**
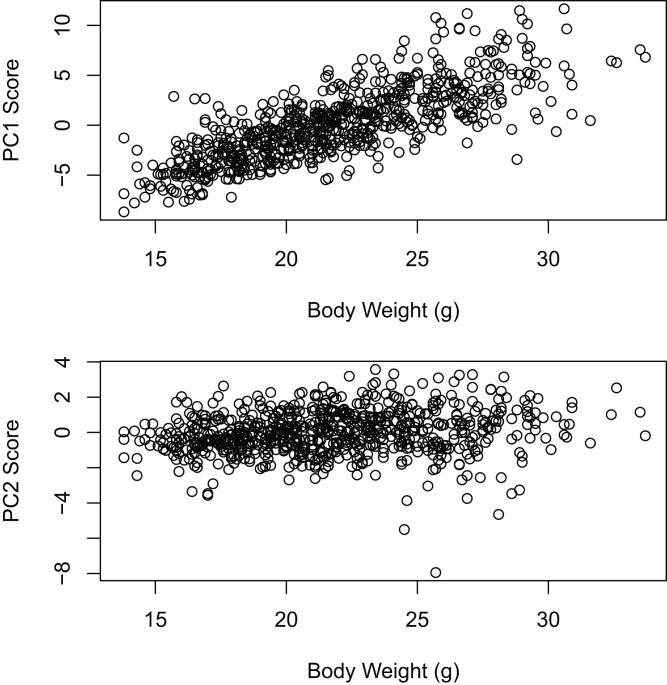
Relationship between principal component (PC) scores and 16-week body weight. Principal components were derived from the pairwise Pearson's correlation matrix of 18 postcranial traits (see *Materials and methods*).

To gain preliminary insights into multivariate patterns among the F2s, we conducted PCA on the pairwise correlation matrix formed from 18 traits (see *Materials and methods*). Principal component 1 (PC1) explains 72% of the phenotypic variance among F2s. The 18 traits included in the PCA make similar contributions to PC1, with loadings ranging from 0.21 to 0.25 across traits. PC1 score is highly correlated with 16-week body weight (Fig. 3; Pearson's *r* = 0.78; *P* < 2.2e−16). Pairwise comparisons with weights taken at other ages (Gray *et al*. 2015) show that the high correlation between PC1 and weight is visible earlier in ontogeny (e.g*. r* = 0.72; *P* < 2.2e−16; 16-week PC1 vs 6-week body weight).

PC2 explains 9% of the phenotypic variance among F2s. Loadings along PC2 vary among traits in both sign and magnitude. The 3 measurements from the ulna have the strongest effects; traits from the scapula and humerus have weak effects. There is a contrast in signs among groups of bones, with increases in the femur and tibia increasing the PC2 score and increases in the ulna and radius decreasing the PC2 score. PC2 score shows a much weaker correlation with 16-week body weight than does PC1 score (Fig. 3; *r* = 0.17; *P* < 4.4e−06).

### QTL for skeletal load resistance traits

**Fig. 4.**
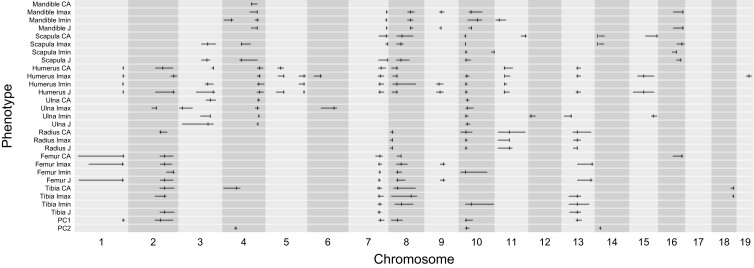
Positions of QTL for bone structure. QTL positions are designated by vertical lines. 1.5 LOD intervals are denoted by horizontal lines.

Single-QTL mapping and multiple-QTL mapping identify QTL for every trait except radius Imin (Supplementary Fig. 1; Table 2; Fig. 4). QTL are detected on every autosome except chromosome 17. The number of detected QTL ranges across phenotypes from 1 (Mandible CA) to 13 (Humerus Imax and Humerus J). Although we conducted no formal tests designed to gauge the extent of shared QTL effects across phenotypes, comparison of QTL positions among traits suggests variation in the breadth of QTL detection (Fig. 4; Supplementary Fig. 1). Some QTL appear to be specific to certain bones, such as QTL located on chromosomes 5 and 19 that are only detected using data from the humerus. Some QTL are detected globally, such as a QTL located on chromosome 10 that impacts every bone surveyed. Most QTLs are detected for a subset of bones. QTL often affect multiple traits, but not always, raising the possibility that resistance measures sometimes have independent genetic bases within the same bone.

**Table 2.** QTL positions and effects.

Bone	Trait	Chr	cM	cM CI	Mb	Mb CI	LOD	PVE	a (SE)	a/SD (SE)	d (SE)	d/a (SE)
Mandible	CA	4	43.78	41.77–53.42	107.44	104.24–128.49	5.20	2.55	0.0112 (0.0068)	0.0779 (0.0472)	0.0451 (0.0099)	4.0411 (2.6309)
Mandible	Imax	4	52.75	38.85–54.46	127.28	99.38–129.35	4.76	2.11	0.007 (0.0059)	0.055 (0.0462)	0.0373 (0.0085)	5.3345 (4.6622)
		7	65.29	62.76–72.66	138.67	137.34–143.58	5.19	2.31	0.0281 (0.0061)	0.2213 (0.0476)	0.0097 (0.0084)	0.3433 (0.3103)
		8	29.94	25.95–35.64	80.87	68.97–95.22	7.67	3.44	0.0325 (0.006)	0.2559 (0.0476)	0.0182 (0.0084)	0.5602 (0.2792)
		9	23.89	20.57–28.66	60.89	54.15–72.98	5.24	2.33	0.026 (0.006)	0.2043 (0.0473)	0.0179 (0.0084)	0.6908 (0.3653)
		10	18.58	14.93–33.22	45.19	36.29–86.34	6.94	3.10	0.0317 (0.0059)	0.2497 (0.0464)	−0.009 (0.0085)	−0.2851 (0.2707)
		16	44.28	25.72–46.4	90.35	54.73–92.45	4.67	2.07	−0.0244 (0.006)	−0.1924 (0.0474)	0.0164 (0.0084)	0.6696 (0.3816)
Mandible	Imin	4	10.36	0.07–13.14	33.23	4.95–39.79	4.62	1.80	−0.0064 (0.0014)	−0.203 (0.0459)	−0.0016 (0.002)	−0.2555 (0.3112)
		4	52.75	46.85–57.63	127.28	118.8–133.75	6.86	2.70	0.0037 (0.0014)	0.116 (0.0443)	0.0095 (0.002)	2.5984 (1.1284)
		7	63.28	60.34–68.81	137.51	134.26–141.31	5.43	2.13	0.0065 (0.0014)	0.2049 (0.0451)	0.0036 (0.002)	0.55 (0.3279)
		8	29.94	25.73–34.08	80.87	68.33–90.97	8.08	3.19	0.0082 (0.0014)	0.2594 (0.0446)	0.0026 (0.002)	0.3198 (0.2463)
		10	27.26	13.14–32.55	68.76	31.59–85.06	10.72	4.26	0.0094 (0.0014)	0.2977 (0.0434)	−0.001 (0.002)	−0.1075 (0.2116)
		11	4.91	0–19.78	16.24	3.31–41.5	5.42	2.12	0.0066 (0.0014)	0.2106 (0.044)	0.0014 (0.002)	0.2113 (0.2969)
Mandible	J	4	52.75	42.29–54.46	127.28	104.69–129.35	5.48	2.37	0.0103 (0.007)	0.0676 (0.0456)	0.0469 (0.01)	4.5491 (3.2359)
		7	65.29	62.76–70.19	138.67	137.34–142.44	5.83	2.53	0.0352 (0.0072)	0.2308 (0.047)	0.0131 (0.01)	0.3717 (0.2952)
		8	29.94	28.48–33.4	80.87	78.56–90.3	8.57	3.74	0.041 (0.0072)	0.2689 (0.047)	0.022 (0.01)	0.5363 (0.2606)
		9	23.89	20.57–23.89	60.73	54.15–60.89	4.77	2.06	0.0287 (0.0071)	0.1879 (0.0467)	0.0221 (0.01)	0.769 (0.4009)
		10	19.29	14.41–19.29	47.01	35.37–47.11	8.11	3.54	0.041 (0.007)	0.2689 (0.0457)	−0.009 (0.01)	−0.2201 (0.2468)
		16	44.28	25.72–46.4	90.35	54.73–92.45	4.87	2.10	−0.0298 (0.0071)	−0.1951 (0.0468)	0.0192 (0.0099)	0.6457 (0.3687)
Scapula	CA	7	63.28	43.58–74.07	137.55	110.12–144.81	4.55	1.75	0.013 (0.003)	0.1918 (0.0448)	−0.0049 (0.0042)	−0.378 (0.3337)
		8	19.77	9.51–32.55	49.10	29.7–90.04	6.76	2.62	0.0156 (0.0029)	0.2299 (0.0425)	−0.0032 (0.0042)	−0.2081 (0.2725)
		10	8.67	6.7–11.73	23.82	20.95–28.13	9.17	3.58	0.0179 (0.0029)	0.2649 (0.0429)	−0.0063 (0.0042)	−0.3498 (0.2391)
		11	62.77	49.99–70.52	111.56	95.55–116.94	5.72	2.21	0.0152 (0.003)	0.224 (0.0449)	0.0001 (0.0042)	0.0084 (0.2756)
		14	2.05	0–13.14	10.01	14.61–35.86	5.64	2.18	0.0145 (0.003)	0.2141 (0.0438)	0.0042 (0.0042)	0.2919 (0.2975)
		15	51.02	18.77–53.33	100.72	57.78–103.09	4.29	1.65	0.0127 (0.0029)	0.1878 (0.0435)	−0.0004 (0.0042)	−0.0316 (0.3315)
Scapula	Imax	3	37.14	30.04–55.06	107.37	83.74–137.52	7.63	2.36	0.0037 (0.0006)	0.2248 (0.0389)	0.0004 (0.0009)	0.1053 (0.2448)
		4	28.14	27.02–40.76	69.04	68.2–103.62	7.47	2.31	0.0035 (0.0006)	0.2144 (0.0388)	−0.0011 (0.0009)	−0.3185 (0.2611)
		7	71.40	60.75–74.07	143.18	134.49–144.81	7.25	2.24	0.0035 (0.0006)	0.214 (0.0387)	−0.0008 (0.0009)	−0.2313 (0.2608)
		8	17.76	9.17–22.04	45.46	28.45–54.12	7.89	2.44	0.0036 (0.0006)	0.2213 (0.038)	−0.0008 (0.0009)	−0.2234 (0.2552)
		10	9.98	7.18–9.98	25.24	22.61–25.42	7.62	2.35	0.0036 (0.0006)	0.223 (0.0387)	0.0004 (0.0009)	0.1183 (0.248)
		14	2.20	0–12.69	9.68	14.61–33.52	4.80	1.47	0.0028 (0.0006)	0.174 (0.0388)	0.0009 (0.0009)	0.3309 (0.3302)
		16	40.10	30.9–50.79	86.64	67.51–96.18	5.36	1.64	−0.0024 (0.0007)	−0.1491 (0.0399)	0.0028 (0.0009)	1.1376 (0.477)
Scapula	Imin	10	10.27	7.69–13.14	26.57	22.61–31.07	19.08	7.72	0.0022 (0.0002)	0.3834 (0.0431)	−0.001 (0.0003)	−0.4491 (0.1671)
		10	66.86	53.46–67.01	129.45	118.68–130.17	5.31	2.06	0.0011 (0.0002)	0.1957 (0.0431)	−0.0006 (0.0004)	−0.5524 (0.3416)
		16	30.53	24.05–30.53	66.59	50.44–66.65	4.51	1.74	0 (0.0002)	−0.0069 (0.0432)	0.0015 (0.0003)	38.8455 (241.87)
Scapula	J	3	35.88	30.26–40.02	104.35	84–115.63	7.72	2.44	0.0047 (0.0008)	0.2265 (0.0391)	0.001 (0.0011)	0.2163 (0.2476)
		4	28.14	24.63–53.42	69.04	62.4–128.49	5.66	1.78	0.004 (0.0008)	0.1916 (0.0392)	−0.0008 (0.0011)	−0.1982 (0.2904)
		7	71.40	43.05–74.07	143.18	108.68–144.81	6.27	1.97	0.0042 (0.0008)	0.2021 (0.0391)	−0.0008 (0.0012)	−0.1803 (0.2771)
		8	17.76	8.47–27.52	45.46	28.29–75.96	6.09	1.92	0.004 (0.0008)	0.1948 (0.0383)	−0.0011 (0.0012)	−0.2659 (0.2935)
		10	10.27	7.18–18.13	26.57	22.61–43.9	12.59	4.04	0.0061 (0.0008)	0.2922 (0.0392)	−0.0006 (0.0012)	−0.0971 (0.1903)
		16	38.65	30.05–38.65	82.67	66.2–84.45	5.89	1.85	−0.0031 (0.0008)	−0.1488 (0.0404)	0.0041 (0.0012)	1.3241 (0.518)
Humerus	CA	1	77.00	74.93–78.67	177.22	175.35–180.7	6.12	2.01	0.0099 (0.0022)	0.1798 (0.04)	−0.0089 (0.0032)	−0.8963 (0.3875)
		2	55.36	46.8–72.69	125.88	98.13–163.66	8.00	2.65	−0.0147 (0.0026)	−0.2659 (0.0463)	0.0007 (0.0033)	0.0491 (0.2226)
		3	47.35	43.48–50.63	127.76	120.96–130.44	4.72	1.55	0.0042 (0.0022)	0.0762 (0.0396)	0.013 (0.0032)	3.0898 (1.7863)
		4	58.85	52.64–68	135.30	126.85–149.31	6.99	2.30	0.0063 (0.0022)	0.1147 (0.0398)	0.0153 (0.0032)	2.4171 (0.9924)
		5	24.93	17.1–29.81	55.35	43.92–66.71	5.67	1.86	−0.0025 (0.0024)	−0.0456 (0.0429)	0.0153 (0.0032)	6.0662 (5.868)
		7	47.83	43.05–61.98	118.74	108.68–136.05	5.29	1.74	0.0105 (0.0023)	0.19 (0.042)	−0.0047 (0.0032)	−0.4509 (0.3176)
		8	9.73	1.32–13.49	30.59	10.52–34.73	9.65	3.21	0.0141 (0.0023)	0.2555 (0.041)	0.0053 (0.0032)	0.3754 (0.2323)
		11	17.47	14.12–35.49	36.19	32.86–66.21	7.73	2.56	0.0131 (0.0023)	0.2373 (0.0414)	−0.0031 (0.0032)	−0.2345 (0.2452)
		13	19.23	17.32–24.06	57.27	54.34–70.38	5.22	1.71	−0.0103 (0.0023)	−0.1858 (0.0413)	−0.0045 (0.0032)	−0.435 (0.3235)
Humerus	Imax	1	77.00	75.58–79.15	177.22	175.35–182.04	7.53	1.99	0.0021 (0.0004)	0.1836 (0.0363)	−0.0018 (0.0006)	−0.8384 (0.3379)
		2	75.12	67.89–89.23	166.22	154.42–178.22	5.07	1.33	−0.002 (0.0004)	−0.1785 (0.0387)	0.0008 (0.0006)	0.3838 (0.2918)
		4	59.71	52.27–64.73	135.90	126–140.41	6.36	1.67	0.0003 (0.0004)	0.0284 (0.0359)	0.0031 (0.0006)	9.4926 (12.1616)
		5	28.99	17.61–30.64	66.71	44.87–68.9	5.96	1.57	−0.001 (0.0005)	−0.0855 (0.0401)	0.0028 (0.0006)	2.8196 (1.4575)
		5	66.91	52.09–74.78	140.66	121.3–147.36	7.30	1.93	−0.0022 (0.0004)	−0.1958 (0.0377)	0.0011 (0.0006)	0.5084 (0.2853)
		6	16.34	5.99–19.69	47.52	23.5–53.55	5.01	1.31	−0.0018 (0.0004)	−0.1601 (0.0366)	0.0006 (0.0006)	0.3449 (0.3435)
		7	45.55	42.83–56.47	116.04	106.6–129.78	5.95	1.56	0.0022 (0.0004)	0.1878 (0.0384)	−0.0007 (0.0006)	−0.3474 (0.2844)
		8	9.96	3.1–12.42	31.02	14.6–33.5	13.32	3.58	0.0032 (0.0004)	0.2765 (0.0368)	0.0007 (0.0006)	0.2073 (0.1898)
		10	12.47	7.69–15.75	29.39	22.61–38.82	13.91	3.75	0.0033 (0.0004)	0.2889 (0.0373)	0.0006 (0.0006)	0.1816 (0.1826)
		11	17.47	15.98–29.73	36.19	35.32–56.97	7.96	2.11	0.0024 (0.0004)	0.2132 (0.0374)	−0.0008 (0.0006)	−0.3435 (0.2513)
		13	19.23	17.32–24.17	57.27	54.34–70.5	7.40	1.95	−0.0023 (0.0004)	−0.2042 (0.0375)	−0.0008 (0.0006)	−0.3463 (0.2611)
		15	16.54	6.81–38.71	52.54	28.9–90.74	5.53	1.45	0.0019 (0.0004)	0.1689 (0.0371)	0.0009 (0.0006)	0.4669 (0.3225)
		19	30.39	22.1–38.82	44.64	33.94–55.04	5.04	1.32	−0.002 (0.0005)	−0.1774 (0.041)	0.0018 (0.0006)	0.8678 (0.3235)
Humerus	Imin	1	76.89	74.28–78	176.39	174.71–179.81	6.83	2.06	0.0015 (0.0003)	0.1879 (0.0382)	−0.0011 (0.0004)	−0.7472 (0.3338)
		3	37.14	33.16–48.06	107.37	96.84–128.59	10.31	3.15	0.002 (0.0003)	0.2589 (0.0386)	0.0001 (0.0004)	0.0509 (0.2107)
		4	57.48	52.27–72.1	133.00	126–153.77	4.86	1.46	0.0008 (0.0003)	0.0978 (0.0382)	0.0017 (0.0004)	2.1834 (1.0338)
		5	66.91	52.09–70.75	140.66	121.3–143.36	10.83	3.31	−0.0019 (0.0003)	−0.2466 (0.0383)	0.001 (0.0004)	0.503 (0.2371)
		7	45.06	43.05–56.47	114.36	108.68–129.78	8.71	2.64	0.0019 (0.0003)	0.2485 (0.0404)	−0.0001 (0.0004)	−0.0773 (0.2212)
		8	9.73	3.1–37.16	30.59	14.6–100.12	11.45	3.50	0.0021 (0.0003)	0.2711 (0.0392)	0.0006 (0.0004)	0.2752 (0.207)
		9	20.18	14.84–26.95	53.14	42.8–69.41	5.50	1.65	0.0011 (0.0003)	0.1398 (0.0395)	0.0015 (0.0004)	1.3424 (0.5514)
		10	10.27	7.18–13.55	26.57	22.61–31.67	12.26	3.76	0.0022 (0.0003)	0.2855 (0.039)	0.0003 (0.0004)	0.1253 (0.1919)
		11	17.47	14.9–22.99	36.19	34.37–45.27	6.56	1.98	0.0013 (0.0003)	0.171 (0.0395)	−0.0013 (0.0004)	−1.0086 (0.3975)
Humerus	J	1	77.00	75.73–78	177.22	175.61–179.81	8.29	2.18	0.0035 (0.0007)	0.1871 (0.036)	−0.0032 (0.001)	−0.9052 (0.3357)
		2	73.52	46.8–80.9	164.04	98.13–170.7	4.79	1.24	−0.0032 (0.0007)	−0.1724 (0.0384)	0.0004 (0.001)	0.1088 (0.3)
		3	47.24	22.06–52.82	127.14	64.68–134.61	6.24	1.63	0.003 (0.0007)	0.1601 (0.0355)	0.0025 (0.001)	0.8394 (0.3759)
		4	59.71	52.12–68.64	135.90	125.51–151.24	5.77	1.50	0.0012 (0.0007)	0.0619 (0.0358)	0.0046 (0.001)	3.9646 (2.4686)
		5	28.99	13.29–30.64	66.71	38.23–68.9	4.72	1.23	−0.0016 (0.0007)	−0.0854 (0.0399)	0.0039 (0.001)	2.42 (1.2849)
		5	66.91	63.65–70.75	140.66	137.17–143.36	9.23	2.43	−0.0042 (0.0007)	−0.2218 (0.0375)	0.002 (0.001)	0.4796 (0.2469)
		7	45.06	43.05–56.47	114.36	108.68–129.78	8.30	2.18	0.0042 (0.0007)	0.2243 (0.038)	−0.001 (0.001)	−0.2337 (0.2325)
		8	9.73	2.43–13.09	30.59	12.23–34.02	13.87	3.71	0.0052 (0.0007)	0.2788 (0.0369)	0.0016 (0.001)	0.3102 (0.19)
		9	21.77	15.21–27.56	57.08	43.14–70.03	4.82	1.25	0.0024 (0.0007)	0.1278 (0.0367)	0.0029 (0.001)	1.2059 (0.5391)
		10	10.27	7.18–13.89	26.57	22.61–33.06	14.69	3.93	0.0055 (0.0007)	0.2922 (0.0367)	0.001 (0.001)	0.191 (0.1777)
		11	17.59	14.12–28.46	36.31	32.86–54.19	7.63	2.00	0.0037 (0.0007)	0.1952 (0.0371)	−0.0023 (0.001)	−0.6153 (0.2908)
		13	19.23	16.2–24.06	57.27	52.06–70.38	5.10	1.33	−0.0032 (0.0007)	−0.1706 (0.0371)	−0.0007 (0.001)	−0.2264 (0.3053)
		15	16.54	2.27–38.48	52.54	12.98–90.61	5.44	1.41	0.0032 (0.0007)	0.1693 (0.0368)	0.0012 (0.001)	0.3818 (0.3151)
Ulna	CA	3	40.31	34.35–55.06	116.47	100.84–137.52	4.88	1.97	0.0064 (0.0014)	0.203 (0.0439)	0.0011 (0.002)	0.1669 (0.3109)
		4	57.48	52.45–60.49	133.00	126.5–136.85	5.01	2.02	−0.0006 (0.0014)	−0.0194 (0.0439)	0.0093 (0.002)	15.1375 (34.4688)
		10	13.10	9.98–16.34	30.90	25.49–39.25	14.15	5.88	0.0112 (0.0014)	0.3555 (0.0445)	0.0002 (0.002)	0.0218 (0.176)
Ulna	Imax	2	47.92	44.16–47.92	102.71	85.08–102.73	5.09	1.64	−0.0009 (0.0002)	−0.2213 (0.0474)	0.0002 (0.0002)	0.2249 (0.2619)
		3	1.48	0–16.48	14.69	3.23–52.4	5.68	1.84	0.0008 (0.0002)	0.1998 (0.0401)	0.0001 (0.0002)	0.1279 (0.2854)
		4	53.42	48.13–58.81	128.49	119.32–135.11	5.99	1.94	−0.0003 (0.0002)	−0.0685 (0.0396)	0.0011 (0.0002)	3.9461 (2.4406)
		6	36.44	16.75–42.7	97.55	49.36–108.37	4.64	1.50	−0.0007 (0.0002)	−0.1739 (0.0391)	−0.0002 (0.0002)	−0.2987 (0.3237)
		10	13.10	9.94–21.08	30.90	24.97–54.02	9.64	3.16	0.001 (0.0002)	0.2604 (0.04)	0.0001 (0.0002)	0.1113 (0.2162)
Ulna	Imin	3	40.31	27.72–40.31	116.47	80.29–116.69	5.55	2.11	0.0002 (0)	0.21 (0.0428)	0 (0.0001)	0.0198 (0.2908)
		4	57.51	55.8–60.49	133.18	131.24–136.85	6.11	2.33	−0.0001 (0)	−0.0981 (0.043)	0.0003 (0.0001)	2.8852 (1.4207)
		10	10.12	7.69–14.89	26.26	22.61–35.62	22.33	8.94	0.0004 (0)	0.4333 (0.0429)	0 (0.0001)	−0.0682 (0.1403)
		12	1.79	0–6.81	9.55	3.61–26.15	4.65	1.76	−0.0002 (0)	−0.1951 (0.0439)	0 (0.0001)	−0.1952 (0.3182)
		13	7.19	0.26–9.2	34.86	8.73–38.28	7.19	2.75	−0.0002 (0)	−0.241 (0.0447)	0.0001 (0.0001)	0.3353 (0.2646)
		15	34.56	30.72–51.32	86.86	79.64–100.92	5.02	1.90	0.0002 (0)	0.2078 (0.0447)	0 (0.0001)	0.1268 (0.2966)
Ulna	J	3	37.52	1.26–48.43	108.42	13.52–129.15	5.74	2.05	0.0009 (0.0002)	0.2042 (0.041)	0.0003 (0.0003)	0.2861 (0.2966)
		4	55.36	51.55–55.36	130.06	125.21–130.07	5.84	2.08	−0.0002 (0.0002)	−0.0499 (0.0412)	0.0013 (0.0003)	5.7959 (4.9688)
		10	13.10	9.94–17.24	30.90	24.97–41.9	14.01	5.12	0.0015 (0.0002)	0.3309 (0.0417)	0.0002 (0.0003)	0.1075 (0.1782)
Radius	CA	2	53.65	51.27–61.65	117.70	112.86–142.06	8.54	3.35	−0.0083 (0.0014)	−0.3051 (0.0501)	0.0016 (0.0017)	0.1885 (0.2038)
		8	3.40	0.12–5.64	14.97	6.93–18.49	7.58	2.97	0.0062 (0.0012)	0.2273 (0.043)	0.0037 (0.0017)	0.6024 (0.2951)
		10	10.12	0–19.85	26.26	7.09–49.73	4.51	1.75	0.0052 (0.0012)	0.1913 (0.0433)	−0.0003 (0.0017)	−0.055 (0.3204)
		11	27.57	3.98–63.51	53.06	11.87–112.28	6.28	2.45	0.0062 (0.0012)	0.2267 (0.0443)	−0.002 (0.0017)	−0.317 (0.2781)
		13	19.23	10.73–44.6	57.27	40.41–108.45	5.17	2.01	−0.0057 (0.0012)	−0.2097 (0.0441)	0.0002 (0.0017)	0.0284 (0.2931)
Radius	Imax	8	3.40	0–3.4	14.68	4.94–14.76	7.23	2.76	0.0004 (0.0001)	0.2271 (0.0424)	0.0002 (0.0001)	0.4434 (0.2817)
		10	10.12	7.69–13.7	26.26	22.61–32.48	14.90	5.82	0.0007 (0.0001)	0.3489 (0.0426)	0 (0.0001)	−0.0256 (0.173)
		11	27.57	2.86–27.57	53.24	11.44–53.65	5.87	2.23	0.0004 (0.0001)	0.2116 (0.0436)	−0.0002 (0.0001)	−0.4413 (0.3008)
		13	19.23	11.88–24.06	57.27	42.18–70.38	7.64	2.92	−0.0005 (0.0001)	−0.2524 (0.0435)	0 (0.0001)	0.0575 (0.2398)
Radius	J	8	3.40	0–3.4	14.68	4.94–14.97	7.31	2.70	0.0007 (0.0001)	0.2286 (0.0417)	0.0003 (0.0002)	0.3544 (0.2716)
		10	10.12	7.69–13.55	26.26	22.61–31.67	16.64	6.33	0.0011 (0.0001)	0.3636 (0.0419)	−0.0001 (0.0002)	−0.0568 (0.1635)
		11	27.57	2.86–34.38	53.61	11.44–64.73	6.16	2.27	0.0007 (0.0001)	0.2186 (0.0429)	−0.0002 (0.0002)	−0.3032 (0.2794)
		13	19.23	11.88–20.67	57.27	42.18–59.19	9.03	3.36	−0.0008 (0.0001)	−0.271 (0.0429)	0 (0.0002)	0.0043 (0.2198)
Femur	CA	1	77.00	2.23–79.15	177.22	12.68–182.04	4.53	1.56	0.0128 (0.0035)	0.1469 (0.0407)	−0.0142 (0.005)	−1.1107 (0.515)
		2	57.93	52.23–74.41	132.63	114.69–164.48	5.13	1.77	−0.018 (0.0041)	−0.2071 (0.0474)	−0.003 (0.0053)	−0.1665 (0.3062)
		7	45.06	41.83–52.78	114.36	98.26–124.69	6.80	2.36	0.0196 (0.0037)	0.2258 (0.0426)	−0.0066 (0.0051)	−0.3372 (0.2656)
		8	17.79	9.66–17.79	45.85	30.26–45.92	10.08	3.54	0.0235 (0.0035)	0.2706 (0.0405)	−0.0014 (0.0051)	−0.0608 (0.2164)
		16	40.10	25.24–45.24	86.64	53.23–90.88	4.83	1.67	−0.0136 (0.0037)	−0.1569 (0.0421)	0.0135 (0.0051)	0.9903 (0.4567)
Femur	Imax	1	75.73	24.76–78	175.61	51.34–179.81	6.05	1.85	0.0057 (0.0013)	0.1687 (0.0382)	−0.0053 (0.0018)	−0.923 (0.3929)
		2	57.93	53.05–70.09	132.63	116.08–160.08	8.19	2.52	−0.0089 (0.0015)	−0.2606 (0.0445)	0.0006 (0.0019)	0.0627 (0.2172)
		7	43.99	43.05–49.99	111.31	108.68–122.11	8.17	2.52	0.008 (0.0014)	0.235 (0.0399)	−0.0021 (0.0019)	−0.2681 (0.2377)
		8	17.79	8.47–26.29	45.85	28.29–69.47	10.78	3.34	0.0089 (0.0013)	0.2629 (0.0381)	0 (0.0019)	−0.0056 (0.2085)
		9	26.61	21.77–30.89	69.13	57.08–76.2	4.77	1.45	0.0051 (0.0013)	0.1505 (0.0387)	0.0045 (0.0019)	0.8872 (0.4323)
		13	47.90	18.62–52.84	111.71	56.94–116.25	5.07	1.55	−0.0061 (0.0013)	−0.1808 (0.0397)	0.003 (0.0019)	0.4894 (0.3175)
Femur	Imin	2	73.52	60.64–78.33	164.04	138.5–169.08	5.16	1.83	−0.0039 (0.0009)	−0.1959 (0.0437)	−0.0009 (0.0012)	−0.2401 (0.3165)
		7	45.06	43.58–47.34	114.36	110.12–117.61	10.44	3.75	0.0059 (0.0009)	0.2918 (0.043)	−0.0006 (0.0012)	−0.1074 (0.2011)
		8	11.19	7.87–19.32	32.34	26.63–48.69	11.74	4.24	0.0059 (0.0008)	0.2919 (0.0405)	0.0005 (0.0012)	0.0777 (0.2029)
		10	9.08	0–42.13	24.40	7.09–104.06	4.46	1.58	0.0036 (0.0008)	0.1797 (0.0409)	0.0005 (0.0012)	0.1427 (0.3261)
Femur	J	1	75.73	4.66–78.04	175.61	14.98–180.01	5.00	1.57	0.0086 (0.0021)	0.1616 (0.0389)	−0.0068 (0.0029)	−0.7917 (0.3939)
		2	57.93	53.05–74.41	132.63	116.08–164.48	7.05	2.23	−0.0131 (0.0024)	−0.2459 (0.0452)	0.0011 (0.0031)	0.0813 (0.2351)
		7	43.99	43.05–47.34	111.31	108.68–117.61	9.32	2.97	0.0135 (0.0021)	0.2545 (0.0404)	−0.0035 (0.0029)	−0.255 (0.2217)
		8	11.60	8.47–23.71	32.48	28.29–61.96	11.05	3.53	0.0143 (0.0021)	0.2689 (0.0386)	0.0012 (0.003)	0.084 (0.2083)
		9	26.61	21.77–30.86	69.13	57.08–75.92	4.85	1.52	0.0082 (0.0021)	0.1547 (0.0393)	0.0072 (0.0029)	0.8741 (0.4231)
		13	43.07	18.62–49.71	107.50	56.94–113.02	4.41	1.38	−0.0095 (0.0022)	−0.179 (0.041)	0.0024 (0.003)	0.2569 (0.3223)
Tibia	CA	2	57.93	51.97–76.89	132.63	114.01–168.31	5.69	1.77	−0.0177 (0.0036)	−0.2217 (0.0451)	0.0022 (0.0046)	0.1257 (0.2548)
		4	19.37	0–26.28	51.92	3.86–65.62	4.58	1.42	0.0138 (0.0031)	0.1735 (0.0393)	−0.0022 (0.0044)	−0.1569 (0.3159)
		7	43.99	42.42–50.51	111.31	103.8–122.29	8.17	2.56	0.0193 (0.0032)	0.2417 (0.0403)	−0.0002 (0.0044)	−0.0103 (0.229)
		8	11.60	4.33–36.75	32.48	17.73–99.19	5.91	1.84	0.015 (0.003)	0.1886 (0.0382)	0.0046 (0.0044)	0.3089 (0.2988)
		18	43.34	35.44–46.65	80.09	69.47–82.15	4.35	1.35	−0.0125 (0.0032)	−0.1571 (0.0397)	−0.0085 (0.0044)	−0.6796 (0.3822)
Tibia	Imax	2	57.93	46.43–61.02	132.63	96.43–139.25	5.92	1.55	−0.004 (0.0008)	−0.21 (0.0413)	0.0012 (0.001)	0.3093 (0.2435)
		7	43.99	42.83–54.37	111.31	106.6–127.73	6.29	1.65	0.0037 (0.0007)	0.194 (0.037)	−0.0002 (0.001)	−0.0603 (0.261)
		8	30.38	1.13–39.43	82.55	9.77–104.42	4.33	1.13	0.0029 (0.0007)	0.1553 (0.0361)	0.0005 (0.001)	0.1796 (0.3277)
		13	19.23	4.35–24.94	57.27	26.18–71.25	4.48	1.17	−0.0029 (0.0007)	−0.1567 (0.0361)	−0.0008 (0.001)	−0.2613 (0.3287)
		18	43.52	39.57–46.65	80.47	75.31–82.15	5.28	1.38	−0.0028 (0.0007)	−0.1486 (0.0367)	−0.0026 (0.0009)	−0.9454 (0.4023)
Tibia	Imin	7	45.06	42.83–50.51	114.36	106.6–122.29	9.05	3.27	0.0033 (0.0005)	0.2736 (0.0433)	−0.0002 (0.0007)	−0.0572 (0.2154)
		8	18.46	6.42–32.55	47.13	22.66–90.04	4.96	1.77	0.0023 (0.0005)	0.1891 (0.0407)	0.0002 (0.0007)	0.0861 (0.3138)
		10	18.80	8.23–65.27	45.81	23.73–127.92	4.49	1.60	0.0022 (0.0005)	0.1832 (0.0415)	−0.0002 (0.0007)	−0.0932 (0.3233)
		13	19.23	4.77–39.83	57.27	26.62–101.17	4.20	1.49	−0.0022 (0.0005)	−0.1796 (0.0421)	−0.0002 (0.0007)	−0.0967 (0.3275)
Tibia	J	2	57.93	52.23–76.89	132.63	114.69–168.31	5.23	1.60	−0.0064 (0.0013)	−0.212 (0.0443)	0.002 (0.0017)	0.3085 (0.2586)
		7	43.99	42.83–48.68	111.31	106.6–119.53	7.28	2.24	0.0067 (0.0012)	0.2229 (0.0396)	−0.0012 (0.0016)	−0.1789 (0.2451)
		13	19.23	5.48–25.48	57.27	29.05–71.5	4.85	1.48	−0.0054 (0.0012)	−0.1786 (0.0388)	−0.0005 (0.0016)	−0.0938 (0.3041)
PC1		1	77.00	72.86–80.91	177.22	173.86–183.37	4.60	1.36	0.5047 (0.1369)	0.1401 (0.038)	−0.5358 (0.1941)	−1.0617 (0.4956)
		2	53.35	46.54–72.69	116.71	96.98–163.66	6.67	1.99	−0.8435 (0.1582)	−0.2342 (0.0439)	0.1298 (0.2028)	0.1539 (0.2343)
		7	46.35	43.05–57.06	117.06	108.68–130.63	6.78	2.02	0.7443 (0.1434)	0.2067 (0.0398)	−0.3245 (0.1984)	−0.436 (0.2797)
		8	11.19	1.13–21.67	32.37	9.77–51.59	8.47	2.54	0.8175 (0.1352)	0.227 (0.0375)	0.1272 (0.1967)	0.1555 (0.2415)
		10	10.27	7.18–20.18	26.57	22.61–51.67	7.82	2.34	0.8093 (0.1383)	0.2247 (0.0384)	0.0118 (0.1938)	0.0146 (0.2395)
		13	19.23	16.2–26.97	57.27	52.06–74.05	4.91	1.46	−0.6428 (0.1399)	−0.1785 (0.0389)	−0.0713 (0.194)	−0.1109 (0.3031)
PC2		4	18.96	14.69–20.94	49.53	43.02–54.34	5.15	2.54	0.219 (0.0619)	0.1741 (0.0492)	−0.2687 (0.0866)	−1.2265 (0.5208)
		10	11.24	7.18–16.34	27.88	22.61–39.25	9.66	4.83	−0.4031 (0.0617)	−0.3204 (0.049)	−0.041 (0.0872)	−0.1018 (0.2169)
		14	5.88	0–5.88	21.52	14.61–21.54	4.80	2.37	−0.2808 (0.0612)	−0.2232 (0.0487)	−0.0157 (0.0882)	−0.0559 (0.3136)

Chr, chromosome; cM, QTL position in cM; CI, 1.5 LOD interval; Mb, QTL position in Mb; LOD, logarithm of odds; PVE, percent F2 variance explained; a/SD, additive effect/F2 standard deviation; d/a, dominance effect/absolute value of additive effect; SE, standard error.

Multiple-QTL mapping identifies 6 QTL that contribute to the PC1 score (Table 2). These QTL are a subset of those found across the collection of traits (Fig. 4). Although QTL for PC1 score map to some of the same chromosomes as QTL for body weight found in the same cross (Gray *et al*. 2015), the 1.5-LOD confidence intervals overlap only for the QTL on chromosome 7. This finding suggests that cross-sectional traits and body weight have distinct genetic architectures, despite their functional relationship. Multiple-QTL mapping identifies 3 QTL that contribute to the PC2 score, one of which overlaps with a QTL for the PC1 score. As a group, QTL for PC1 and QTL for PC2 have narrower 1.5-LOD intervals than most measurements mapped on the original scale.

To further examine the breadth of QTL effects, we compared the fit of a null model assuming a single QTL affects a set of traits to the fit of models assuming 2 linked QTL affect the same set of traits. We focused on 3 QTL with overlapping confidence intervals across many traits: a region on chromosome 7 linked to 18 traits, a region on chromosome 8 linked to 20 traits, and a region on chromosome 10 linked to 17 traits. For the regions on chromosomes 7 and 8, we failed to reject the null hypothesis of a single QTL (*P* > 0.05). For the region on chromosome 10, we rejected the null hypothesis of a single QTL (*P* = 0.01). Two QTL separated by 5.9 cM are detected, with the most proximal QTL (at 11.35 cM) affecting 14 postcranial traits, and the other QTL (at 17.24 cM) modulating mandible Imax, mandible Imin, and mandible J. These results support the notion that some QTL impact resistance abilities across many dimensions and bones, though the power to reject the null hypothesis in favor of multiple closely linked QTL is constrained by the sample size and the number of crossovers in an F2.

**Fig. 5.**
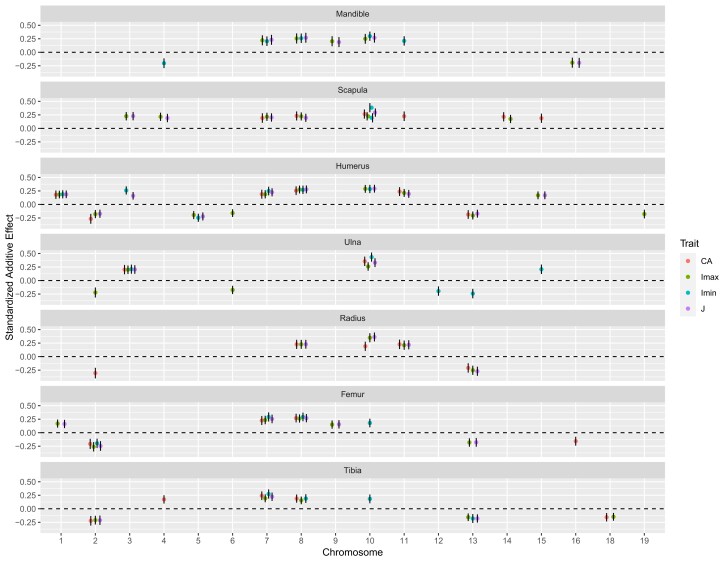
Phenotypic effects of bone structure QTL. Additive effects divided by F2 phenotypic standard deviations are shown. Traits are distinguished by colors. Points within bones and chromosomes are randomly separated for visual clarity (depicted positions are unrelated to actual positions along chromosomes). Vertical lines display ± 1.96 SEs. Dashed line denotes 0. QTL with |*d/a*| > 1 are excluded.

Standardized additive effects of QTL are summarized in Fig. 5 and Table 2. Across QTL (excluding those mapped for principal component scores and those with absolute scaled dominance effects (|*d/a*|) > 1), the absolute values of additive effects range from 0.149 to 0.433 F2 phenotypic standard deviations, with a mean of 0.223. Among these QTL, 93 have positive additive effects, indicating that the GI mouse allele increases the trait value, whereas 37 have negative additive effects, indicating that the GI mouse allele decreases the trait value. Each bone features QTL with a mixture of positive and negative additive effects. ANOVA treating bone and trait (CA, Imax, Imin, and J) as fixed effect factors (again excluding QTL with |*d/a*| > 1) finds evidence for differences in standardized additive effect absolute values among bones (F_6,120_ = 2.93; *P* = 0.01) and among traits (F_3,120_ = 2.73; *P* = 0.05). A Tukey post hoc test indicates that tibia-radius (*P* = 0.02), ulna-tibia (*P* = 0.03), and Imin-Imax (*P* = 0.03) contrasts contribute to the significant differences in additive effects among bones and among traits.

Eighty-five percent of QTL (130 of 153) have scaled dominance effects (*d*/*a*) that fall below 1 in absolute value (median *d*/*a* = 0.31) (Table 2), revealing relatively weak dominance overall. An exception is a QTL region on distal chromosome 4, which has high *d/a* values for multiple traits on the mandible, humerus, and ulna (Table 2). When all QTL are considered, scaled dominance effects do not differ among bones (F_6,143_ = 0.81; *P* = 0.56) or among traits (F_3,143_ = 0.49; *P* = 0.69). When QTL with |*d/a*| > 1 are excluded, scaled dominance effects differ among bones (F_6,120_ = 3.61; *P* = 0.003) but not among traits (F_3,120_ = 2.03; *P* = 0.11).

The percent variance among F2s explained by individual QTL ranges from 1.13 to 8.94% (Table 2), with a mean of 2.40%.

### Candidate genes for skeletal load resistance

We focused our search for candidate genes on the shared QTL on chromosomes 10 and 7, which affect 14 postcranial traits and 18 traits (respectively) and have 1.5-LOD intervals that span less than 10 Mb (Table 3). *Ccn2* is the only gene located in the chromosome 10 shared QTL interval that is known to play a specific role in the formation of the skeleton. Phenotypes of knockout mice demonstrate that *Ccn2* (also known as *Ctgf*) regulates extracellular matrix remodeling that affects endochondral bone ossification (Ivkovic *et al*. 2003) (though it should be noted that long bone in adults is mostly produced by intramembranous ossification). *Ccn2* harbors a single nonsynonymous SNP between the parents of our cross. The variant is located at amino acid position 1 in one transcript and amino acid position 24 in a second transcript. In one transcript, the GI variant constitutes an alternative start codon that encodes threonine instead of methionine. This substitution is predicted to be deleterious (SIFT score = 0.01).

**Table 3.** Candidate genes for QTL that affect the structures of several bones.

Gene symbol	Gene name	Gough AA	WSB AA	AA position	SIFT score	Chr	QTL cM	QTL interval cM	QTL Mb	QTL interval Mb
*Ccn2*	Cellular communication network factor 2	Threonine	Methionine	1 or 24	0.01 or 0.6	10	11.35	8.23–11.73	27.94	23.73–28.13
*Scube2*	Signal peptide, CUB domain, EGF-like 2	Methionine	Isoleucine	145	1	7	44.91	43.05–47.38	114.21	108.68–117.83
*Scube2*	Signal peptide, CUB domain, EGF-like 2	Valine	Isoleucine	417	1	7	44.91	43.05–47.38	114.21	108.68–117.83
*Spon1*	Spondin 1, (f-spondin) extracellular matrix protein	Arginine	Glutamine	102	0.9	7	44.91	43.05–47.38	114.21	108.68–117.83
*Pth*	Parathyroid hormone	—	—	—	—	7	44.91	43.05–47.38	114.21	108.68–117.83
*Xylt1*	Xylosyltransferase 1	—	—	—	—	7	44.91	43.05–47.38	114.21	108.68–117.83

AA, amino acid; Chr, chromosome; —, no amino acid difference.

The shared QTL region on chromosome 7 contains several genes with established effects on skeletal morphology (Table 3). *Scube2* mouse mutants show reduced osteoblast cell number (Lin *et al*. 2015). *Scube2* contains 2 nonsynonymous SNPs between the parents of our cross. Both SNPs have SIFT scores of 1, predicting no deleterious consequences. Knockout mice for *Spon1* exhibit increased bone mass in the femur and tibia (Palmer *et al*. 2014). The single nonsynonymous SNP between the parents of our cross found in *Spon1* is predicted to be tolerated (SIFT score = 0.9). Long bones from *Pth* knockout mice have higher cortical thickness (Miao *et al*. 2002). *Xylt1* regulates early chondrocyte maturation in mice (Mis *et al*. 2014).

## Discussion

Our findings point to several conclusions about the evolution and genetics of determinants of bone strength. GI mice expanded bone cross-sectional area and increased moments of inertia without enhancing bone density. This result suggests that the increased skeletal loads imposed by extra weight can be mitigated by structural changes without altering the material properties of bone in an evolutionary context.

Structural determinants of load resistance abilities evolved in a coordinated fashion across the bones of GI mice. Correlations among traits in F2s suggest particularly tight integration between changes to the femur and changes to the tibia/humerus, which could reflect shared developmental trajectories between these bones. Although it is difficult to distinguish between pleiotropy and linkage as explanations for the colocalization of QTL in an F2 intercross, overlapping confidence intervals and mapping of principal component scores imply that several QTL alter the structure of multiple bones. Loci on chromosomes 7, 8, and 10 affect a plethora of traits throughout the skeleton.

At the same time, structural QTL vary in their breadth of detection. Some QTL appear restricted to single bones and some QTL were detected for certain traits but not others within the same bone. We caution that a full picture of the distribution of shared QTL effects across bones and traits awaits analyses that consider QTL effects in a multivariate context. Nevertheless, these findings significantly expand the view of the genetics of load resistance in the mouse skeleton beyond previous investigations, which focus primarily on the femur. Our results underscore the need for genetic studies to consider multiple bones.

We detected no QTL that alter bone structure in major ways, despite the precipitous evolution of body size in GI mice. All QTL additive effects are smaller than 0.44 F2 standard deviations and each QTL explains less than 9% of F2 variance, despite an expected upward bias in effect size estimates for detected QTL (Beavis 1994). Therefore, the path to increased load resistance abilities in GI mice followed expectations for typical quantitative traits, involving many mutations with individually modest effects. A similar genetic inference has been drawn for the evolution of other morphological traits in GI mice (Gray *et al*. 2015; Parmenter *et al*. 2016, 2022).

Parmenter *et al*. (2016) report QTL for bone length and width in the same cross we analyzed here. Most QTL from the 2 studies do not overlap, indicating that the evolution of cortical structure and the evolution of whole-bone measures were mostly accomplished by different genes. A subset of bone structure QTL overlap with those from Parmenter *et al*. (2016), including QTL on chromosome 7 for humerus CA, humerus Imax, humerus Imin, and humerus J (humerus midshaft diameter) and for tibia CA, tibia Imax, tibia Imin, and tibia J (tibia midshaft diameter); QTL on chromosome 2 for femur CA, femur Imax, and femur J (femur length) and for femur Imin (femur midshaft diameter); and QTL on chromosome 10 for tibia CA (tibia length and proximal tibia width). The shared QTL on chromosomes 7 and 2 are also recovered when mapping PC1 in the current study, suggesting they could be determinants of bone morphology across the skeleton.

Several caveats accompany our conclusions. Direct measurement of bone strength ultimately requires mechanical tests (Adams and Ackert-Bicknell 2015), which we did not conduct. Although biomechanical models and empirical comparisons in mice connect the CA and moments of inertia we analyzed with load resistance (Turner *et al*. 2001; Wergedal *et al*. 2006; Jepsen 2009; Jepsen *et al*. 2015), these traits still represent indirect measures of performance. More realistically, cross-sectional measurements capture load resistance abilities rather than performance abilities used by mice as part of their behaviors. Mapping bone strength estimated by mechanical tests could yield different QTL. Nevertheless, our results provide genetic insights into the evolution of determinants of bone strength relevant to load resistance.

Another limitation of our study is its restriction to a single time point, 16 weeks of age. Based on the developmental dynamics of bone structure, we might expect the underlying genetic architecture to depend on age. For example, femur geometry changes with age, and peak bone strength is not reached until 20 weeks in mice from one classical inbred strain (Brodt *et al*. 1999).

A final caveat is that our evolutionary interpretation is constrained by a lack of knowledge about the most appropriate strain to compare to GI mice. For a mainland reference strain, we used WSB, a wild-derived inbred strain developed by breeding mice trapped in Maryland. Mice are thought to have colonized GI from Western Europe, but the geographic source has proven difficult to resolve (Gray *et al*. 2014). More generally, some QTL we identified could represent mutations that accumulated along the lineage leading to WSB mice rather than mutations along the lineage leading to GI mice. This inability to polarize QTL is an inherent limitation of all genetic mapping studies that only consider 2 strains.

Our investigation establishes GI mice as a tractable genetic system for understanding how vertebrates evolve to resist loads in their skeletons. Our QTL maps constitute the first step toward identifying specific genes and mutations responsible for the evolution of bone structure in island organisms with unusually large bodies. With the genetic tools available for house mice, the GI mice system is poised to deliver insights into the molecular, cellular, and developmental mechanisms that enable expansions of body size in nature.

## Supplementary Material

iyad137_Supplementary_Data

## Data Availability

F2 phenotypic data (“F2_phenotypes.xls”) and genotypic data (“goughF2_simple_v4.Rdata”) are available through GSA figshare: https://doi.org/10.25386/genetics.22194526. Supplemental material available at GENETICS online.
