# Supplementary material for: Genetics of evolved load resistance in the skeletons of unusually large mice from Gough Island
Source: Genetics. 2023 Jul 21;225(1):iyad137. doi: 10.1093/genetics/iyad137 (PMC10471205; doi:10.1093/genetics/iyad137)

Figure S1. LOD plots from single-QTL scans. Y-axis = LOD score. X-axis = chromosome. Horizontal lines denote significance thresholds from permutations.

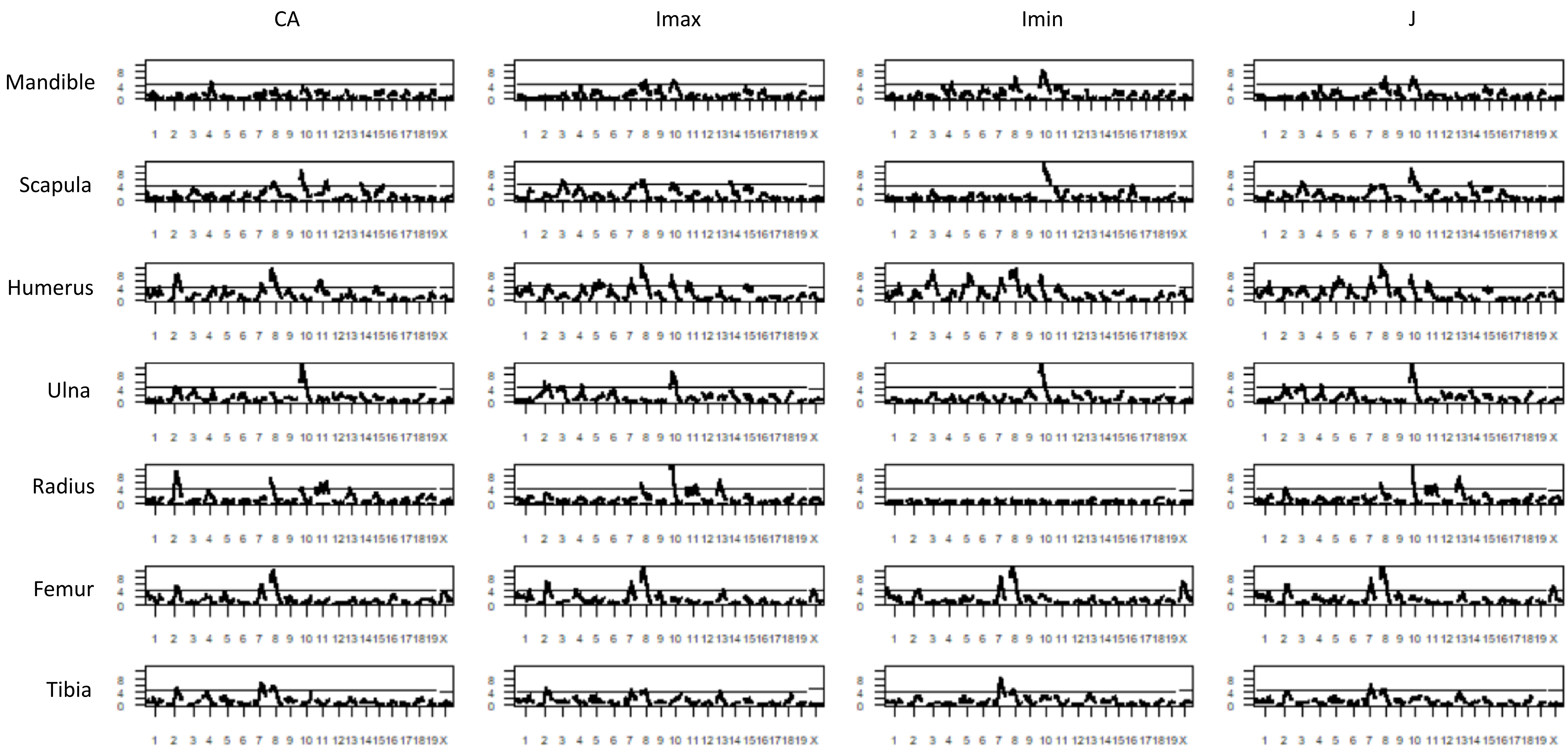

Supplement: iyad137_Supplementary_Data [file iyad137_supplementary_data.pdf]
